# An ethnobotanical study of medicinal plants in Wayu Tuka District, East Welega Zone of Oromia Regional State, West Ethiopia

**DOI:** 10.1186/1746-4269-9-68

**Published:** 2013-09-25

**Authors:** Moa Megersa, Zemede Asfaw, Ensermu Kelbessa, Abebe Beyene, Bizuneh Woldeab

**Affiliations:** 1 Department of Biology, Medawalabu University, P.O. Box 247, Robe, Ethiopia; 2 National Herbarium, Addis Ababa University, P.O. Box 3434, Addis Ababa, Ethiopia; 3 Department of Environmental Health Sciences and Technology, Jimma University, P.O. Box 378, Jimma, Ethiopia

## Abstract

**Background:**

This paper reports an ethnobotanical study that focused on the traditional medicinal plants used by local communities to treat human and livestock ailments. A cross-sectional study was undertaken from September 2009 to June 2010 in Wayu Tuka District of Oromia Region, Ethiopia. The aim of the study is to document medicinal plants used by local people of the study area and the threats currently affecting medicinal plants.

**Methods:**

Ethnobotanical data were collected using semi-structured interviews, field observations and group discussion in which 63 (41 men & 22 women) randomly selected informants participated. Of which, 11 (10 male and 1 female) were local healers. Paired comparison method, direct matrix ranking and Informant consensus factors (ICF) were used to analyze the importance of some plant species.

**Results:**

A total of 126 medicinal plant species, distributed in 108 genera and 56 families, were collected together with their medicinal uses. Of the 126 species of medicinal plants collected from the study area, eighty six (68%) were obtained from the wild whereas thirty three (26%) were from homegardens. The Fabaceae came out as a leading family with 15 medicinal species while the Solanaceae followed with eight species. Seventy eight (62%) of the medicinal plants were reported as being used for treating human ailments, 23 (18.2%) for the treatment of livestock ailments and 25 (20%) for both. The most frequently used plant parts were leaves (43%), followed by roots (18.5%) while crushing, which accounted for (29%) and powdering (28%) were the widely used methods of preparation of traditional herbal medicines.

**Conclusion:**

The number of reported medicinal plants and their uses by the local people of the District indicate the depth of the local indigenous knowledge on medicinal plants and their application. The documented medicinal plants can serve as a basis for future investigation of modern drug.

## Background

Since time immemorial, people have used plants as medicine. The investigation of plants and their uses is one of the most primary human concerns and has been practiced by all cultures for tens, if not hundreds, of thousands of years, though it wasn’t called ‘Ethnobotany’ [1]. Perhaps as early as Neanderthal humans, plants were believed to have healing powers [2]. The earliest recorded uses are found in Babylon about 1770 BC and in the code of Hamurabian ancient Egypt about 1550 BC. In the early 1500 s, Indian fever bark was one of the first medicinal plants to find appreciative consumers in Europe, which was taken from the cinchona tree (*Cinchona officinalis*), the bark of which was used as an infusion by native people of the Andes and Amazon highlands to treat fevers. Jesuit missionaries brought the bark to Europe and by the early 16^th^ century the name of this medicine was transformed to “Jesuit fever bark” [2].

Traditional medicine comprises of therapeutic practices that have been in existence, for hundreds of years, before the development and spread of modern medicine and are in use today [3]. These practices vary widely, in keeping with the social and cultural heritage of different countries. Traditional medicine includes a diversity of health practices, approaches, knowledge, and beliefs incorporating plant, animal, and/or mineral-based medicines; spiritual therapies; manual techniques; and exercises, applied singly or in combination to maintain well-being, as well as to treat, diagnose, or prevent illness [4]. Traditional medicine was once again redefined in 2008 as the sum total of knowledge, skills and practices based on the theories, beliefs and experiences indigenous to different cultures that are used to maintain health, as well as to prevent, diagnose, improve or treat physical and mental illnesses [5].

A major component of traditional medicine is that which uses medicinal plants. Plant-based traditional medicine plays a key role in the development and advancement of modern studies by serving as a starting point for the development of novelties in drug discovery [6]. Various modern drugs were extracted from traditional medicinal plants through the use of plant material following the ethnobotanical leads from indigenous cures used by traditional medical systems [7]. On top of their use in fighting various ailments at local level, different medicinal plants are used as export commodities, which generate considerable income [8]. China takes the lead (45%) by importing the highest number of herbal medicines for preparation of drugs and this is followed by the United States of America (15.6%) and Australia (10.5%) [9].

In Ethiopia, the use of traditional medicinal plants is widely practiced. The wide spread use of traditional medicine in Ethiopia could be attributed to cultural acceptability, efficacy against certain type of diseases, physical accessibility and economic affordability as compared to modern medicine [10]. The size of the Ethiopian flora is estimated at 6,000 species of vascular plants of which about 10% are believed to be endemic [11-18]. Traditional remedies are the most important and sometimes the only source of therapeutics for nearly 80% of the Ethiopian population and 95% of the preparations are of plant origin [10]. Due to various reasons, such as knowledgeable people in the society, the knowledge on medicinal plants of the country is getting lost. Since the knowledge of traditional medicine is transferred orally from generation to generation, basic information on the use of the plants and the part used, drug preparation methods, the diseases treated and others may be lost and discarded in the knowledge transfer process. Therefore, documentation of medicinal plants and the indigenous wisdom associated with them is important in order to pass the knowledge to the next generation since the plant materials and the indigenous knowledge can be the basis for the invention of modern drugs on top of the heritage values of the resource. In addition, such studies are vital in order to identify threatened medicinal plant species to give due attention for proper management and conservation. Thus, this study was initiated to document the traditional medicinal plant knowledge of the people and the threats currently affecting medicinal plants in Wayu Tuka District.

## Materials and methods

### Study area and the people

**Figure 1:**
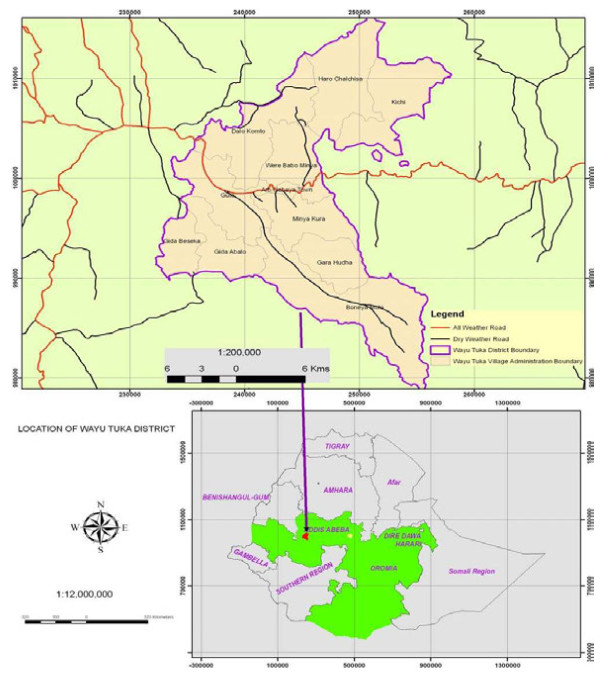
Location of Wayu Tuka District in East Welega Zone; Oromia Regional State.

Wayu Tuka District is situated at (8° 56′N and 9° 7′N) and (36° 32′E and 36° 48′E). It is located at about 322 km west of Addis Ababa, in the East Welega Zone of the Oromia National Regional State. The District covers an area of 28,952.795 ha and comprises 12 kebeles (smallest administrative unit) belonging to ten rural areas and two urban centers namely ‘Boneya Molo’, ‘Gara Hudha’, ‘Gute Badya’, ‘Kichi’, ‘Komto’, ‘Migna Kura’, ‘Haro Chalchis’, ‘Gida Abalo’, ‘Gida Basaka’, ‘Wara Babo Miya’, ‘Gaba Jimata’ and ‘Gute’ (Figure 1).

According to Wayu Tuka District Agricultural Office [19], the altitude of the study area ranges from 1300–3140 m.a.s.l, and the District has various topographic features. About 17,950.8445 ha (62%) of the land area is plain, 4,922.00575 ha (17%) hilly, and mountains and clifts account for 3,763.88675 ha (13%) and 2,316.238 ha (8%) respectively. The major soil types are clay loom, covering about 17371.68 ha (60%), sandy soil that stretches over an area of 10133.49 (35%) and clay constitutes 1447.64 ha (5%). The latter two soil types are suitable for agriculture including for cultivation of cereal crops including maize (*Zea mays*), sorghum (*Sorghum bicolor*) and ‘teff’ (*Eragrostis tef*) [19].

**Figure 2:**
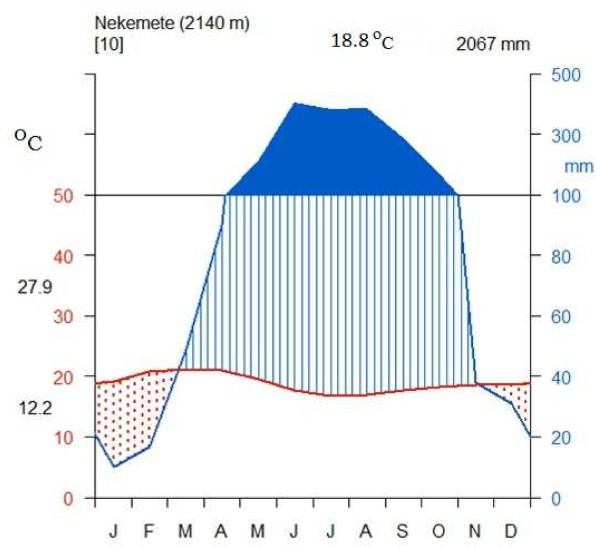
**Climadiagram of the study area from 1998–2007 at Nekemte Weather Station, East Welega Zone.** Data source: National Meteorological Service Agency.

Based on the metrological data recorded at Nekemte station for 10 years (1998–2007), the rainfall distribution of the district is unimodial. The rainy season is locally called ‘Ganna’ and it extends from May to August with the highest peak in June and August. The highest average monthly rainfall was recorded in June (4,026.7 mm) and the lowest in January (99.9 mm), with the hottest months from March to October. The maximum mean temperature was recorded in February and March (27.9°C) and the coldest months of the year stretch from November to January, the lowest temperature having been recorded in December and January (12.2°C). In general, the mean annual temperature and mean annual rainfall of the District are 18.8°C and 2,067 mm, respectively (Figure 2).

The vegetation of the area belongs to the moist evergreen montane forest and this type of forest is known to occur in southwest Ethiopia, particularly in parts of Welega, Ilubabor and Kefa [20]. The common species in the area include *Poutenia adolfi-friederici, Trilepisium madagascariense, Morus mesozygia, Mimusops kummel, Podocarpus falcatus, Coffea arabica* and *Galiniera saxifraga.*

Based on the 2007 population and housing census, the population of Wayu Tuka District is projected to be 66394, with 63325 (95.4%) in the rural, directly living on agriculture and assocciated activities also by supplying its produce to the neighboring urban dwellers. The people of the District belong to the Oromo ethnic community. Afaan Oromo (the Oromo language) is the widely spoken language in the area.

The District has three governmental clinics, four governmental health posts and one non-governmental health post. In the District, the leading ten human diseases are internal parasites (intestinal), rheumatism, upper respiratory diseases, skin diseases, diarrhea, malaria, gastritis, and fever of unknown causes, ear diseases and anemia [*Wayu Tuqa Wereda Health Office:* Report on diseases found in the Wereda, Unpublished].

There are two veterinary clinics in the District where the number of cattle and the number of clinics are not balanced. The ten most serious livestock diseases in the District are trypanasomiasis, internal parasites, external parasites, pasteurollosis (ovine and bovine), blackleg, anthrax, African horse sickness, sheep and goat pox, New Castle disease, babeosis and mastits [19].

### Methods

A reconnaissance survey of the study area was carried out from September 15 to 30, 2009 and resulted in the identification of nine study sites, namely Boneya Molo, Gaba Jimata, Gara Hudha, Gute, Gute Badya, Kichi, Komto, Migna Kura and Wara Babo Miya. The study sites were selected based on the availability of practice of traditional medicine, and on the recommendations of elders and local authorities in Wayu Tuka District. Moreover, the three agro-climatic zones were also considered to select the study sites (kebeles).

### Ethnobotanical data collection

A total of 63 (41 males and 22 females) informants were selected out of 66394 population following [21]; 43 were selected randomly and 20 key informants were selected purposively and systematically based on the recommendations of knowledgeable elders, local authorities and development agents by taking 2–3 individuals from each study site. Out of which 11 were traditional healers (10 males and 1 female). The informants were local inhabitants aged between 19–102 years. The selection of key informants was also based on the quality of explanations that particular informants gave during an interview. Local healers automatically qualified as key informants being traditional experts who are custodians of indigenous knowledge on medicinal plants.

Ethnobotanical investigations were carried out to collect data on medicinal plants used to treat human and livestock ailments in Wayu Tuka District following standard methods [21,22]. The techniques used were semi-structured interviews, field observations, group discussion and guided field walk. The data were collected from October 1, 2009 to December 15, 2009 and March 26, 2010 to April 06, 2010. Interviews and discussions were undertaken based on checklist of questions prepared in English and translated to ‘Afaan Oromo’. Information was carefully recorded during an interview with an informant as well the knowledge of vegetation categorization was asked and recorded. Field observations were performed with the help of local guides on the morphological features and habitats of each medicinal plant species in the field.

Discussions were conducted on threats to medicinal plants, conservation of the medicinal plants and transferability of knowledge in the community. Before collecting the data, written permission was secured from the office of the District and permission was obtained from the administrator of each selected kebele. Following this, the purpose of the study was explained to each informant and verbal prior consent was obtained.

### Specimen collection and identification

The reported medicinal plants were collected from natural vegetation and homegardens during the field walks and habits of the plants were listed. Preliminary identification was done at the site (field) and the collected voucher specimens were taken to the National Herbarium of Ethiopia (Addis Ababa University). Specimen identification and confirmation was undertaken by using taxonomic keys and various volumes of the Flora of Ethiopia and Eritrea [11-18]. Finally, the identified specimens were reconfirmed by a taxonomic expert and the specimens with their label stored at the National Herbarium.

### Data analysis

The collected ethnobotanical data were entered into Excel spreadsheet 2007 and summarized using descriptive statistical methods such as frequency and percentages. Paired comparison method [21] was used to determine the relative importance of plant species, which are used in the treatment of blackleg. In paired comparison, 9 informants were selected and asked to choose the best item from every pair according to personal perception in treating Blackleg. The total number of possible pairs (21) was obtained by applying the formula **n (n-1)/2**, where **n** is the number of medicinal plants being compared. A total rank of paired comparison was obtained by summing the number of times each item was chosen. An item with highest frequency of choices had the highest score.

Direct matrix ranking [21,22] exercises were employed in order to compare the multiple uses of a given plant species based on information gathered from informants. The multipurpose species were selected out of the total medicinal plants and the uses of these plants were listed and 8 randomly selected key informants were asked to assign use values to each species. Each chosen key informant was asked to assign use values (5 = best, 4 = very good, 3 = good, 2 = less used, 1 = least used and 0 = not used). The values (average scores) of each species were summed up and ranked.

The Informant consensus factor (ICF) was calculated for each category to identify the agreements of the informants on reported cures for the group of ailments. The ICF [23] was calculated as follows

ICF=nur−nt/nur−1

Where,

ICF=InformantsConsensusFactor

nur=numberofusecitationineachcategory

nt=numberofspeciesused

## Results

### Indigenous knowledge and local vegetation categories

People of the study area classify vegetation of their surroundings mainly based on density of plant species that cover the land. The following four categories of vegetation were used by the community to distinguish one vegetation type from another. They then describe the location of a medicinal or other useful plant distribution in terms of these categories.

‘**Caffee’** is marshy vegetation where mostly plant species of the families Poaceae and Cyperaceae grew. The place is generally considered unsuitable for ploughing and crop cultivation but is suitable for grazing.

‘**Luugoo-lagaa’** is equivalent to reverine vegetation which is found at the banks of rivers, mostly composed of *Syzygium guineense* subsp. *guineense* and *Ficus sycomorus.*

**‘Bosona’** is a type of forest with densely populated plant species with many tall trees, making the home of wild animals. An example of such vegetation in the study area is ‘Bosona Komto’ (Komto Forest), which is found in Komto Kebele.

**‘Daggala’** is the term used to refer to seasonal plants.

### Medicinal plants of the study area

One hundred twenty six species, belonging to 108 genera and 56 families, were used by local people of the District to treat various human and livestock ailments (Tables 1, 2 and 3**).** There were seven endemic species of Ethiopia found among the reported traditional medicinal plants (*Albizia malacophylla*, *Coccinia abyssinica*, *Impatiens tinctoria* subsp. *abyssinica*, *Lippia adoensis*, *Pycnostachys abyssinica* and *Saturegia paradoxa*)*.* Among the families that contributed more medicinal species were the Fabaceae, represented by 15 species (12%), Solanaceae with 8 (6.3%) species, Asteraceae with 7 (5.6%), and other 44 families contributing 57 (45%) species are represented by 1 or 2 species (Table 4). Of the 126 species of medicinal plants collected from the study area, most of them (86, 68%) were obtained from the wild whereas 33 (26%) were from homegardens, and only 7 (5.5%) species were from both homegardens and wild habitats **(**Tables 1, 2 and 3**).**

**Table 1:** List of medicinal plants for treating human diseases in the study area, Wayu Tuka District

**Scientific name**	**Local Oromo name**	**Family**	**Hab**	**Ha**	**Plant part, preparation and application**	**Disease treated**	**V. No.**
*Acacia abyssinica* Hochst. ex Benth.	Laaftoo	Fabaceae	T	W	Juvenile leaves crushed and sniffed	Bat urine	MM072
*Acmella caulirhiza* Del.	Guutichaa	Asteraceae	H	Hg	Fresh flowers chewed and swallowed	Tonsillitis	MM038
*Albizia gummiffera* (J. F. Gmel.) C.A. Sm.	Muka arbaa	Fabaceae	T	W	Leaves crushed, mixed in water. Put in cotton and rubbed on affected teeth.	Toothache	MM068
					Bark chewed in order to get relief from Rheumatism	Rheumatism	
*Albizia* sp.	Ambaltaa	Fabaceae	T	W	Dried bark powdered and applied on affected part	Wound	
							MM077
*Allium sativum L.*	Qullubbii adii	Alliaceae	H	Hg	The bulb taken with ‘injera’ and Capsicum annuum L. for 5 days before eating breakfast	Malaria	MM013
*Asparagus africanus Lam.*	Sariitii	Asparagaceae	Sh	W	Fresh leaves crushed and applied on the affected part	Spider poison	MM092
*Bidens macroptera (Sch. Bip. ex Chiov.) Mesfin*	Keelloo	Asteraceae	H	W	Fresh leaves put on fire and rubbed on affected part	Athletes foot	MM037
*Brassica carinata* A. Br.	Goommana	Brassicaceae	H	Hg	Dried seed Powdered and mixed with water then drunk.	Common cold	MM002
*Brucea antidysentrica* J.F. Mill.	Qomanyoo	Simaroubaceae	T	B	Fresh leaves crushed and mixed with Leaves of *Bersema abyssinica* Fresen. and cooked With porridge and given for a person in need	Ascaris	MM028
					Root powdered and mixed in water and drunk	Diarrhea	
*Croton macrostachyus* Del.	Bakkanisa	Euphorbiaceae	T	W	Exudates put on the cut skin to stop bleeding	Skin cut	MM080
					Bark of croton put on fire and the smoke used as to protect mosquito bite	Mosquito repellant	
					Juvenile leaves smashed and rubbed on affected part	Ring worm	
					Dried root powdered and given to Dog with ‘injera’ which suffered by Rabies	Rabies	
*Catha edulis* (Vahl) Forssk ex Endl.	Caatii	Celastraceae	T	Hg	Fresh leaves crushed and boiled in water with leaves of *Ruta chalepensis* L.*,* fresh leaves of *Periploca linearifolia* Quart. –Dill. & A. Rich. and fresh leaves of *Englerina woodfordioides* Gilbert then sugar added while it is boiling, put off from the fire and make to cool finally a cup of tea will be taken for four days.	Cough	MM020
*Carissa spinarum* L.	Agamsa	Apocynaceae	Sh	W	Fresh bark chewed early before having breakfast	Stomach ache	MM103
					The bark Chewed or hold in teeth for 5-10 min.	Toothache	
*Canarina eminii* Aschers ex Schweinf.	Maaracaa	Campanulaceae	Cl	W	The whole plant crushed together, chewed and swallowed	Headache	MM154
					Whole plants crushed and rubbed on affected part	Scabies	
*Capparis tomentosa* Lam.	H.gurraacha	Capparidaceae	Sh	W	Roots crushed and sniffed	Fibril illness	MM101
*Carica papaya* L.	Paappaayyaa	Caricaceae	T	Hg	When the leaves become yellow, that means getting to dry, powdered and boiled in water and a cup of tea will be taken for 5 days.	Malaria	MM085
					The steam crushed and tied on affected part	Wound	
					Seed chewed and swallowed	Internal parasite	
*Caylusea abyssinica* (Fresen.) Fisch. and Mey.	Illancoo	Residaceae	H	W	Fresh leaves cooked and eaten with ‘injera’ /bread	Amoeba	MM110
*Centella asiatica* (L.) Urban	Baala buqqee	Apiaceae	H	W	Leaves crushed and rubbed	Tinea corporis	MM058
*Citrus limon* (L.) Burn.f.	Loomii	Rutaceae	T	Hg	Squiz the fruit and massage on bleeding gum	Gum bleeding	MM022
					Crush the fruit and apply its content on skin burn.	Skinburn	
*Citrus aurantium* L.	Qomxaaxxee	Rutaceae	T	Hg	Suck the content of the fruit when suffered by hypertension	Hypertension	MM021
*Clausena anisata* (Wild.) Benth	Ulmaayii	Rutaceae	Sh	W	Leaves powdered and mixed with water and given immediately for the victimed	Snake bite	MM090
					Bark of *Clausena anisata,* leaves of *Sida rhombifolia,* root of *Cucumis ficifolius,* bark root of *Brucea antidysentrica* powdered together and mixed in milk then drunk a cup of tea for three days in order to get cured from Rabies disease	Rabies	
*Clematis simensis* Fresen.	Hidda fiitii	Ranunculaceae	Cl	W	Fresh root chewed	Stomach ache	MM198
*Clutia abyssinica* Joub. & Spach.	Ulee foonii	Euphorbiaceae	Sh	W	Fresh leaves hold in teeth for 20–30 minutes	Toothache	MM098
*Coffea arabica* L.	Buna	Rubiaceae	Sh	Hg	The dried coffee bean roasted and powdered then given to the patient by mixing with honey.	Diarrhea	MM017
*Coccinia abyssinica* (Lam.) Cogn.	Ancootee	Cucurbitaceae	H	Hg	The root Cooked with leaves of *Croton macrostachyus* and eaten with ‘injera’ for *four* days.	Tuber closes	MM100
*Cordia africana* Lam.	Waddeessa	Boraginaceae	T	W	Leaves of *Cordia africana,* leaves *of Acanthus polystachius* crushed together with Feces of goat then put on fire the ash mixed with butter and creamed on affected part.	Spider poison	MM091
*Crotalaria spinosa* Hochst. ex. Benth.	Shumburaa gugee	Fabaceae	H	W	Root crushed, mixed with water and drunk	Rabies	MM067
*Cymbopogon citratus***(**DC.) Stapf	Marga citaa	Poaceae	H	Hg	Fresh root chewed with salt to get relief from stomach ache	Stomach ache	MM173
*Cynoglossum lanceolatum* Forssk.	Maxxannee	Boraginaceae	H	W	Fresh leaves smashed and the exudates dropped in ear	Ear disease	MM124
					Fresh Leaves crushed and sniffed	Headache	
					Leaves smashed and the extracts dropped in eye	Eye disease	
					Leaves powdered with leaves of *Croton macrostachyus* and creamed on the affected part by mixing with butter	Homeroide	
*Datura strumanium* L.	Asaangira	Solanaceae	Sh	W	Fresh leaves smashed and smelled	Nasal bleeding	MM084
					Seed put on fire and the smoke inhaled	Tooth ache	
*Drynaria volkensii* Heiron.	Baala balleessaa	Polypodiaceae	Ep	B	Fresh root put on fire and until get hot and then bite by affected teeth for an hour	Tooth ache	MM059
*Echinops hispidus* Fresen.	Keberchoo	Asteraceae	H	W	Dried bark put on fire and the smoke inhaled	Evil eye	MM034
*Ehretia cymosa* Thonn.	Ulaagaa	Boraginaceae	T	W	Fresh leaves chewed	Toothache	MM009
*Embelia schimperi* Vatke	Hanquu	Myrsinaceae	Li	W	Fruit eaten early in the morning	Tape worm	MM047
*Ensete ventricossum Cheesman*	Baala warqee	Musaceae	H	Hg	The latex half cup of tea taken to get relief from stomach ache	Stomach ache	MM012
*Eucalyptus globulus* Labill	Akaakltii adii	Myrtaceae	T	B	Fresh leaves boiled in water and then the patient laid down in it in order to inhale the smoke	Common cold	MM087
*Euphorbia tirucalli* L.	Cadaa	Euphorbiaceae	Sh	Hg	The milky latex dropped on affected part	Homeroide	MM005
*Gardenia ternifolia* Schumach.	Gambeela	Rubiaceae	T	W	Fresh seed put in fire ad when it gets hot put on affected part	Homeroide	MM019
*Grewia ferruginea* Hochst. ex A. Rich.	Dhoqonuu	Tiliaceae	T	W	The hair washed by leaves of *Grewia ferruginea* and used as a soap	Dandruff	MM048
*Hagenia abyssinica* (Brace) J.F.Gmel.	Heexoo	Rosaceae	T	W	The dried or fresh floral part powdered soaked in water and left for four days and taken with coffee before having break fast	Tape worm	MM089
*Indigofera arrecta* Hochst. ex A.Rich	Heennaa	Fabaceae	Sh	W	Leaves powdered and mixed with butter and applied on the affected part for five days	Spider poison	MM078
*Indigofera spicata* Forssk.	Reencii	Fabaceae	H	W	Leaves powdered and mixed in water and taken when need arise.	Diabetics	MM203
*Lagenaria siceraria* (Molina) Standl.	Buqqee seexanaa	Cucurbitaceae	H	W	Put on fire and burn the affected part	Dandruff	MM099
*Leucas martinicensis* (Jacq) R.Br.	Fidoo	Lamiaceae	H	W	Steam put on fire and let the patient laid in it for smoke	Eye disease	MM065
*Lippia adoensis* Hochst. ex Walp	Kusaayee	Verbenaceae	H	B	Fresh leaves chewed	Burn on chest	MM109
*Mirabilis jalapa* L.	Ababa diimaa	Nyctagnaceae	Sh	W	Creamy powder of the fruit will be rubbed on affected part.	Homeroide	MM111
*Momordica foetida* Schumach.	Humbaawoo	Cucurbitaceae	H	W	Root washed, crushed and mixed with water and the exudates taken for five days one liter per a day.	Kidney problem	MM029
*Ocimum urticifolium Roth*	Ancabbii	Lamiaceae	Sh	Hg	Fresh leaves crushed and smashed then the extracts rubbed on affected part	Fibril illness	MM133
*Olea europaea* L*.* subsp. *cuspidata* (Wall. ex G. Don) Cif.	Ejersa	Oleaceae	T	W	Fresh root chewed	Stomach ache	MM041
*Panicum hochstetteri* Steud.	Marga gogorrii	Poaceae	H	W	Fresh leaves chewed	Kidney problem	MM127
*Pavonia urens* Cav.	Hincinnii	Malvaceae	H	W	Powdered leaves tied on affected part	Wound	MM062
*Phytolacca dodecandra* L’ Herit.	Andoodee	Phytolaccaceae	Li.	Hg	Few root powdered and mixed with water and drunk for two days	Gonorrhea	MM088
					Root of *Phytolacca dodecandra,* juvenile leaves of *Momordica foetida* leaves of *Justicia schimperiana* and juvenile leaves *of Croton macrostachyus powdered* together and very few given with tea before having breakfast for three days*.* One cup of tea is given for man whereas half cup of tea for children	Liver disease	
*Plantago lanceolata* L.	Qorxobbii	Plantaginaceae	H	W	Fresh leaves crushed and tied	Skin cut	MM044
*Plectranthus edulis* (Vatke) Agnew	Dinnicha oromoo	Solanaceae	Sh	W	Root cooked and eaten	Loss of appetite	MM108
*Prunus africana* (Hook. f.) Kalkm.	Hoomii	Rosaceae	T	W	Powdered and tied for five days	Wound	MM016
*Prunus persica* (L.) Batsch	Kookii	Rosaceae	T	Hg	Juvenile leaves dried and powdered then mixed with butter and creamed on affected part in Wednesday and Friday	Tinea corporis	MM007
*Pycnostachys abyssinica* Fresen.	Yeeroo	Lamiaceae	H	W	Fresh leaves crushed, smashed and the extracts dropped in the eye	Eye disease	MM129
*Rhamnus prinoides* L Herit.	Geeshoo	Rhamnaceae	Sh	Hg	Fresh leaves chewed	Tonsillitis	MM081
*Ricinus communis* L.	Qobboo	Euphorbiaceae	H	B	Fresh leaves crushed and mixed with water and taken one cup of tea for 3 consecutive days.	Rabies	MM006
					Fresh root crushed and mixed with root of *Justicia schimperiana* and put in cup of tea and mixed with water and drunk	Liver disease	
*Rumex abyssinicus* Jacq.	Dhangaggoo	Polygonaceae	H	W	Leaves crushed and smashed then applied on affected part	Scabies	MM053
*Ruta chalepensis* L*.*	Ciraaddama	Rutaceae	H	Hg	Fresh leaves and roots chewed	S.ache	MM083
*Rytigynia neglecta***(**Hiern) Robyns	Mixoo	Rubiaceae	T	H	Leaves powdered and sniffed	Bat urine	MM056
*Saccharum officinarum* L.	Shankora	Poaceae	Sh	W	Steam put in fire and eaten when get hot in order to get relief from common cold	Common cold	MM093
*Schinus molle* L.	Qundoobarbaree	Anacardaceae	T	W	Fresh seed chewed	Tonsillitis	MM023
*Saturega paradoxa* (Vatke) Engl. ex Seybold	Kefo sa’aa	Lamiaceae	H	W	Fresh leaves crushed and sniffed	Bat urine	MM200
*Securidaca longepedunculata* Fresen.	Xamanaayii	Polygalaceae	T	W	Dried roots crushed and put on fire then the smoke sniffed	Evil eye	MM112
					Dried bark powdered and taken with local alcohol for 5 days	Liver disease	
*Senna septemtrionalis* (Viv) Irwin & Barneby	*Samamakii*	Fabaceae	Sh	W	Fresh leaves smashed and mixed with water then one cup of tea taken.	Snake bite	MM145
*Stephania abyssinica* (Dillon & A. Rich.) Walp.	Hidda kalaalaa	Mensipermaceae	H	W	The whole part of *Stephania abyssinica* crushed and boiled in water then the smoke will be inhaled until the patient getting sweat	Common cold	MM040
*Solanum gigantum* Jacq.	Hiddii saree	Solanaceae	Sh	W	Root crushed and taken with coffee	Rabies	MM202
*Solanum incanum* L.	Hidi lonii	Solanaceae	Sh	W	Break the fruit and drop its content on wound to stop bleeding	Wound	MM118
*Vernonia auriculifera* Hiern	Reejii	Asteraceae	Sh	W	Fresh leaves smashed and the extracts dropped on the cut skin	Skin cut	MM033
*Vicia faba* L.	Baaqelaa	Fabaceae	H	Hg	Dried seed chewed	Gastric	MM003
*Vigna unguiculata* (L.) Walp.	Hiphoo	Fabaceae	Cl	Hg	Fresh leaves smashed and rubbed on affected part	Tinea corporis	MM063
*Vigna vexillata* L. A. Rich.	Gurra hantuutaa	Fabaceae	Cl	W	Leaves crushed with leaves of *Cucumis ficifolius* A. Rich. and rubbed on affected part	Spider poison	MM066
*Oliverella hildebrandtii* (Engl.) Tieghem	Dheertuu dhumugaa	Loranthaceae	Ep	Hg	Fresh leaves crushed and rubbed on hair	Dandruff	MM 031
*Ximenia americana* L.	Hudhaa	Olacaceae	Sh	W	Crushed and mixed with water and one cup of tea taken for 1–5 days until the blood stop	Menstruation	MM152
					Exudates drunk for five days 2 cup per a day.	Contraceptive	
*Zingiber officinale* Roscoe	Jinjibila	Zingebraceae	H	Hg	Chewed and swallowed	Tonsillitis	MM011

Key: *Hab* Habit: *H* Herb, *Sh* Shrub, *T* Tree, *Cl* Climber and *Li* Liana; *Ha* habitat: *W* Wild, *Hg* Homegarden, *B* Both, *V. No.* Voucher number.

**Table 2:** List of medicinal plants for treating livestock diseases in the study area, Wayu Tuka District

**Scientific name**	**Local Oromo name**	**Family**	**Hab**	**Ha**	**Plant part, preparation and application**	**Disease treated**	**V. No.**
*Acacia persiciflora* Pax	Garbii	Fabaceae	T	W	The powdered bark mixed in water and given for the cattle forcefully	Stomach ache	MMO64
*Acanthus polystachius* Delile	Kosorruu	Achantaceae	Sh	W	Fresh leaves crushed and rubbed on affected part (wound)	‘Madaa gatiittii’	MM106
*Albizia malcophylla* (A. Rich.) Walp.	*Arganboobee*	Fabaceae	T	W	Bark powdered and given for treatment of Blackleg	Blackleg	MM070
*Buddleja polystachya* Fresen.	Hanfaarree	Loganaceae	Sh	W	Fresh leaves smashed and the extracts dropped in the eyes of affected cattle	Eye disease	MM024
*Colocasia esculenta* (L.) Schott	Goodarree	Araceae	H	B	Tuber crushed and mixed with water then given to the cow	Delayed placenta	MM027
*Combretum collinum* Fresen.	Unuunuu	Combretaceae	T	W	A bottle of mixed fresh crushed bark given for cattle by one bottle forcefully	Breast ulcer	MM102
*Combretum molle* R. Br. ex. G. Don	Dabaqqaa	Combretaceae	T	W	Steam put on the fire and rubbed the affected tongue	Tongue infection	MM149
*Girardinia bullosa* (Steud.) Wedd.	Gurgubbee	Urticaceae	Sh	W	Root powdered and mixed in water and applied orally	Blackleg	MM046
*Grewia bicolor* Juss.	Harooressa	Tiliaceae	T	W	Bark of *Grewia bicolor* grinded and mixed in water and salt added finally given for the cattle which placenta is delayed during delivery	Delayed placenta	MM151
*Guizotia scabra* (Vios.) Chiov.	Tuufoo	Asteraceae	H	W	Fresh leaves of *Guizotia scabra* and leaves of *Calpurnia aurea* crushed and rubbed	External Parasite/silmii	MM191
*Helinus mystacinus* (Ait.) E. Mey. ex Steud.	Hidda hoomoo	Euphorbiaceae	Cl	W	Leaves crushed and smashed and rubbed for external parasite	External parasite	MM097
*Hymenodictyon floribundum* (Hochst. & Steud.) Robinson	Altadhahaa	Rubiaceae	T	W	Fresh leaves smashed and the exudates dropped in the eyes of affected cattle	Eye disease	MM156
*Impatiens tinctoria* A. Rich. subsp. *abyssinica* (Hook. f. ) Grey Wilson	Qicuu	Balsaminaceae	H	W	Powdered root taken	Blackleg	MM153
*Rhus ruspolii* Engl.	Xaaxessaa	Anacardaceae	T	W	Fresh leaves crushed and rubbed on affected part	External parasite	MM051
					Root of *Rhus ruspolii* Engl. powdered and mixed with water and drunk	Hyena bite	
*Solanum anguivi* Lam.	Hidii seexanaa	Solanaceae	Sh	W	Fresh fruit boiled in water and dropped in the eyes of affected cattle	Eye disease	MM120
					Root grinded and mixed with water and given One bottle for three days	Trypanosomiasis	
*Sorghum bicolor* (L.) Moench	Bisingaa caabbii	Poaceae	H	Hg	Seed mixed in remnants of local beer and given	Delayed placenta	MM094
*Thalictrum rhynchocarpum* Dill. & A. Rich.	Mararree	Rananculaceae	H	W	The whole part crushed and given	Blackleg	MM045
*Teclea nobilis* Del.	Hadheessa	Rutaceae	Sh	W	Leaves crushed and mixed with water and given for the thin cattle	Thinness	MM025
					Steam powdered and mixed with water and given forcefully by beer bottle	Anthrax	
*Verbascum sinaiticum* Benth.	Gurra harree	Scrophulariaceae	H	W	Fresh leaves powdered and mixed in water then given orally for external parasite	External parasite	MM125

Key: *Hab* Habit: *H* Herb, *Sh* Shrub, *T* Tree, *Cl* Climber and *Li* Liana, *Ha* habitat, *W* Wild, *Hg* Homegarden, *B* Both, *V. No.* Voucher number.

**Table 3:** List of medicinal plants for treating both human and livestock diseases in the study area, Wayu Tuka District

**Scientific name**	**Local Oromo name**	**Family**	**Hab**	**Ha**	**Plant part, preparation and application**	**Disease treated**	**V. No.**
*Calpurnia aurea* (Ait.) Benth.	Ceekaa	Fabaceae	Sh	W	Fresh leaves soaked in water and wash the body of calf	External parasite	MM074
					9 juvenile leaves of *Calpurnia aurea,* 9 leaves of *Senna occidentalis* and 9 juvenile leaves of *Clausena anisata* smashed and the extracts taken. One cup of tea is given for man and half cup for Children	Ascaris	
					Leaves crushed and mixed in water given by bottle forcefully	Snake bite	
*Cucurbita pepo* L*.*	Buqqee	Cucurbitaceae	H	Hg	The dried seed roasted and eaten	Tape worm	MM018
					Fruit cooked and rubbed on affected part	External parasite	
*Cucumis ficifolius* A. Rich.	Faca’aa	Cucurbitaceae	H	W	Very few fresh root chewed with salt	Gonorrhea	MM026
					Very few root powdered and mixed with one litter of water then given to the cattle forcefully	Blackleg	
*Guizotia abyssinica* (L.f.) Cass.	Nuugii	Asteraceae	H	Hg	Seed roasted powdered and the decoction drunk	Swelling	MM036
					Seed powdered and rubbed on madaa gatiitii of oxen	Madaa gatiittii	
*Hordeum vulgare* L.	Garbuu	Poaceae	H	Hg	Seed of *Hordeum vulgare* powdered with seed of *Brassica carinata* and drunk	Swelling	MM082
					Seed covered and left to let germinate then grinded and mixed with remnants of local beer or ‘tella’ and given orally	Blotting	
*Justicia schimperiana* (Hochst. ex Nees) T. Anders.	Dhummuugaa	Acanthaceae	Sh	Hg	Fresh leaves crushed and given for hen or cock	Coccidiosis	MM008
					Leaves put on fire with leaves of *Brucea antidysentrica* and rubbed on head	Headache	
*Lepidium sativum* L.	Shinfaa	Brassicaceae	H	Hg	Dried seed powdered and eaten with injera to get cure from malaria or rubbed the body for protection from mosquito bite	Malaria	MM015
					Seed powdered in water and given by the bottle forcefully	Blackleg	
*Linum usitatissimum* L.	Talbaa	Linaceae	H	Hg	The hair washed by seeds of *Linum usitatissimum* and used as a soap	Dandruff	MM096
					Seed powdered and given by mixing in water	Breast ulcer	
*Lotus corniculatus* L.	Abbaa qiddii	Fabaceae	H	W	Powdered root taken with tea	Snake bite	MM065
*Kalanchoe laciniata* (L.) DC.	Bosoqqee	Crassulaceae	H	W	Fresh or dried root of *Kalanchoe laciniata,* seed of *Capsicum frutescens, Allium sativum* and leaves of *Croton macrostachyus* Powdered together and given for affected cattle	Blackleg	MM158
*Malva verticillata* L.	Karfichoo	Malvaceae	H	W	Leaves cooked and the smoke inhaled to get relief from ‘Mich’	Fibril illness	MM150
*Maesa lanceolata* Forssk.	Abbayyii	Myrsinaceae	Sh	W	Fresh leaves crushed and rubbed on the body	External parasite	MM079
*Nicotiana tabacum* L.	Tamboo	Solanaceae	H	Hg	Leaves crushed and mixed with water and drunk	Snake bite	MM004
					Leaves crushed and tied on affected part	Snake poison	
					Leaves crushed and put in the mouth then the cow will not drink water or feed for certain minutes until the leech come out	Leeching	
*Rumex nepalensis* Spreng.	Timijjii	Polygonaceae	H	W	Few root chewed and swallowed	Gastric	MM055
					Fresh leaves crushed and mixed with Leaves of *Acanthus polystachius* By mixing with butter creamed on affected part	Spider poison	
					Root powdered and mixed in water then mixed in water and given for the cattle forcefully(waga’uu)	Blackleg	
*Stereospermum kunthianum* Cham.	Botoroo	Bignoniaceae	T	W	Fresh/ Dried bark of *Stereospermum kunthianum* Cham., bark of *Croton macrostachyus,* Root of *Cucumis f*icifolius*,* bulb of *Allium sativum* L*.* and seed of *Capsicum frutescens* powdered together and half of a bottle given for three days	‘Kaashmeer’	MM176
					Dried bark put on fire and the smoke inhaled	Evil eye	
					Powdered and mixed with water and one cup of tea taken for three days	S.ache	
					Leaves crushed and rubbed	Spider poison	
*Vernonia amygdalina* Del.	Eebicha	Asteraceae	Sh	Hg	Leaves crushed and mixed with remnants of local beer(‘Tella’)and given for the cow	Delayed placenta	MM010
					Leaves crushed and soak in water and the exudates drunk orally for five days	Malaria	

Key: *Hab* Habit, *H* Herb, *Sh* Shrub, *T* Tree, *Cl* Climber and *Li* Liana, *Ha* habitat, *W* Wild, *Hg* Homegarden, *B* Both, *V. No.* Voucher number.

**Table 4:** Taxonomic diversity of medicinal plants in the study area

**Family**	**Number of genera**	**Percentage**	**Number of species**	**Percentage of species**
Fabaceae	11	10.0	15	12.0
Solanaceae	4	3.7	8	6.3
Asteraceae	5	4.6	7	5.6
Lamiaceae	5	4.6	6	4.7
Poaceae	6	5.5	6	4.7
Cucurbitaceae	5	4.6	5	4.0
Rutaceae	4	3.7	5	4.0
Euphorbiaceae	5	4.6	4	3.0
Rubiaceae	3	2.7	4	3.0
Boraginaceae	3	2.7	3	2.3
Malvaceae	3	2.7	3	2.3
Rosaceae	2	2.0	3	2.3
Other 44 families	52	48.0	57	45.0
Total	108	100	126	100

**Figure 3:**
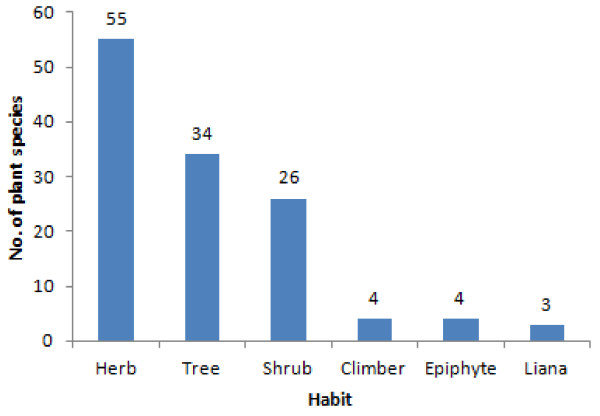
**Growth forms (habits) of medicinal plants in the study area**.

The result of growth form analysis of medicinal plants showed that herbs constituted the highest proportion being represented by 55 (43.6%) species, while there were 34 (27%) tree species, 26 (20.6%) shrubs and 3 (2%) lianas (Figure 3).

**Figure 4:**
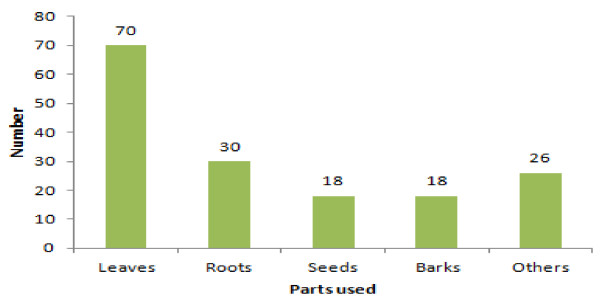
Plant parts used for the treatment of human and livestock ailments.

Informants of the study area harvest different plant parts for preparation of traditional drugs (e.g. leaves, roots, seeds, barks and fruit). In the study area, the informants reported that more species (70, 43%) of medicinal plants were harvested for their leaves and these were followed by roots (30, 18.5%), seed and bark (18, 22.2%) each and 26 others (bulb, tuber, stem, fruit and flower) covered 16% (Figure 4).

Among the collected 126 medicinal plant species, 78 (62%) were claimed to treat human health problems (Table 1), 23 (18.2%) were claimed to treat livestock ailments (Table 2) and 25 (20%) were for both human and livestock ailments (Table 3)**.**

People of the study area mostly administer traditional medicine orally. This accounted for 64%, followed by dermal administration (27.3%) and others (nasal, anal, optical, ear) accounting to 8.3%. Local people also reported that various additives were given during administration of traditional medicine.

### Condition, dosage and effectiveness of traditional medicine in the study area

The majority of the remedies (74.2%) in the study area were prepared from fresh parts of medicinal plants followed by dried form (20.7%) and (5%) prepared either from dry or fresh plant parts. Most of the medicinal plant preparations involved the use of single plant species or a single plant part (85%) while those mixing different plants or plant parts (15%) were rarely encountered in the study area. Healers, usually prepare remedies by mixing various plants or plant parts. Lack of consistency regarding amount of medicines to be used was observed among informants during the interview.

Local people of the study area used various ways of measuring dosage which were generally categorized under three major classes. One is dosage used for those medicinal plants which are expected to be highly toxic. For such medicines the measurement was undertaken by little finger index and very few amounts of the prepared medicine taken by a cup of coffee (Locally known as ‘Sinii’). For example, medicines prepared from *Phytolacca dodecandra, Cucumis ficifolius, Carissa spinarum* and *Securidaca longepedunculata* are toxic if overdosed*.* The second is the dosage used for medicinal plants which can have little effect. The dosage is measured by hand palm and taken by bottle or locally made material from *Lagenaria siceraria* known as ‘Hullee’. E.g., traditional medicines prepared from *Vernonia amygdalina*. In the third case there are medicinal plants that do not have any observable side effect. Medicines prepared from *Allium sativum, Citrus limon,* and *Citrus aurantium* can be taken according to personal preference of the patient. Moreover, informants indicated the effectiveness of traditional medicines to get relief from certain diseases including rabies and health problems associated with the liver, spider poisoning and those caused by bat urine.

### Methods of preparation of traditional medicine

In the study area, the most common methods of preparation of traditional medicine from plant material was crushing (29%), followed by powdering (28%) and others (Table 5).

**Table 5:** Method of preparation of traditional medicine in the study area

**Method of preparation**	**Number of preparations**	**Percentage**
Crushing	39	29.0
Powdering	38	28.0
Chewing	19	14.0
Concoction	17	12.6
Decoction	11	8.0
Others	13	10.0

### Importance of medicinal plants in the study area

Paired comparison ranking of 7 medicinal plants that were reported as effective for treating blackleg, was conducted after selecting 9 informants. The informants were asked to compare the given medicinal plants based on their efficacy. The results showed that *Cucumis ficifolius* scored the highest mark and ranked first indicating that it was the most effective in treating blackleg and followed by *Lepidium sativum* (Table 6).

**Table 6:** Paired comparison of medicinal plants used to treat blackleg in the study area

**Medicinal plants used**	**Respondents (R1-R9)**										
	R1	R2	R3	R4	R5	R6	R7	R8	R9	Total	Rank
*Clematis simensis*	1	1	1	1	1	0	1	0	1	7	6^th^
*Cucumis ficifolius*	5	5	5	4	6	4	6	4	5	44	1^st^
*Cyphostemma cyphopetalum*	0	1	1	0	0	2	0	1	0	5	7^th^
*Kalanchoe laciniata*	3	4	2	2	4	3	2	5	2	27	5^th^
*Lepidium sativum*	3	4	5	5	3	4	5	4	5	38	2^nd^
*Rumex nepalensis*	3	3	4	4	4	4	4	5	4	35	3^rd^
*Thalictrum rhynchocarpum*	6	3	3	5	3	4	3	2	4	33	4^th^

### Direct matrix ranking of multipurpose medicinal plants

Among the medicinal plants reported by the informants, there were those that were used for other purposes and thus grouped as multipurpose species. Key informants first identified eight medicinal plant species that were used by the community for additional purpose including fire wood, charcoal making, construction purposes, food, fencing and forage. Application of direct matrix ranking to these species showed that *Cordia africana* was the best, followed by *Eucalyptus globulus* and *Crotom macrostachyus* (Table 7).

**Table 7:** Direct matrix ranking of eight multipurpose medicinal plants (Average score of 8 key informants)

**Plant species**	**Use categories**							**Total**	**Rank**
	**Medicine**	**Fire wood**	**Charcoal**	**Construction**	**Food**	**Fence**	**Forage**		
*Acacia abyssinica*	2	4	5	4	0	2	0	17	4^th^
*Cordia africana*	3	4	5	5	3	2	0	22	1^st^
*Croton macrostachyus*	5	4	3	4	0	2	0	18	3^rd^
*Eucalyptus globulus*	4	5	3	5	0	2	0	19	2^nd^
*Justicia schimperiana*	5	2	0	1	0	4	3	15	6^th^
*Prunus africana*	2	3	4	4	0	3	0	16	5^th^
*Rhamnus prinoides*	4	1	1	0	0	3	3	12	7^th^
*Ricinus communis*	3	1	0	0	2	3	1	10	8^th^

### Informant consensus factor (ICF)

The result showed that, diseases that were frequent in the study area have higher Informant Consensus Factor. Medicinal plants that are effective in treating certain disease and well known by community members also have higher ICF. Malaria and headache had the highest ICF value (0.85) whereas, Rabies had the lowest ICF value (0.25) (Table 8).

**Table 8:** Informant consensus factor by categories of diseases in the study area, Wayu Tuka District

**Category**	**No. of spp.**	**Total % of spp.**	**No. of use citation**	**% of use citation**	**ICF**
Malaria and headache	7	5.5	42	13.0	0.85
Fibril illness, swelling and evil eye	9	7.0	40	12.4	0.79
Intestinal parasite, diarrhea, amoeba and stomach ache	17	13.5	60	19.0	0.72
Ear, eye and tooth ache (Organ)	7	5.5	23	7.5	0.71
Cattle ailments (Blackleg, Anthrax, Leech and External parasite)	14	11.0	30	10.0	0.55
Common cold and cough	8	6.0	16	5.0	0.53
Snake bite, spider poison and bat poison	16	12.6	28	9.0	0.44
Skin diseases, skin cut and wound	25	20.0	41	13.0	0.40
Lung, kidney and liver diseases (Organ)	8	6.0	12	4.0	0.36
Gonorrhea and menstruation	3	2.0	4	2.0	0.33
Rabies	7	5.5	9	3.0	0.25

### Threats to medicinal plants and conservation practices in the study area

In Wayu Tuka District various factors that were considered as main threats for medicinal plants were recorded by discussion with the informants. Accordingly, the major factors reported were deforestation for the purpose of agricultural expansion (75%), overgrazing (10%), collection of plant material for construction (10%) and fire wood (5%).

People of the study area know the benefits of conserving medicinal plants. However, the effort of conserving medicinal plants is very limited (minimal). That is an evident for being only 26% of medicinal plants were collected from homegarden. Local healers who frequently make use of medicinal plants for a living do not conserve medicinal plants very well, and they preferred to collect them from wild stands when patients visit them. It was explained by informants that local healers do this in order not to let the other community members know the identity of the medicinal plants they are using. Informants further explained that if healers planted the species in their homegardens, they suspect that somebody else might see them while they are preparing the medicine from the plants and start to prepare them and reduce the income which could have gone to the healer. Further observations showed some medicinal plants frequently growing in homegardens, including *Ocimum urticifolium* and *Ruta chalepensis,* the medicinal plant knowledge of which is in the public domain**.** Beliefs have reported to have some contributions to conservation of medicinal plants. It was reported that medicinal plants collected during ‘Chegino’ (that means Monday, Wednesday and Friday) are not used, and limitation of days for collecting medicinal plants reduces the effect of over-harvesting.

## Discussion

### Medicinal plants used to treat human and livestock ailments in the study area

A considerable number (126) of medicinal plants have been documented in this study. The number of reported medicinal plants and their uses by the local people of the District indicates the depth of the local indigenous knowledge on the medicinal plants and their applications. Out of the collected medicinal plants, 78 species were reported for use in the treatment of human diseases, whereas 23 species were used to treat livestock ailments and 25 species were used to treat both human and livestock ailments. Similar findings were reported by other studies [24-27] in other parts of Ethiopia where local people use more medicinal plants to treat human diseases than livestock ailments.

Various studies [27-29] conducted in Ethiopia as well as in other countries of the world reported that the majority of medicinal plants are being harvested from non-cultivated areas. This observation is a good indication of the fact that the local people have not yet started cultivating the majority of the plant species they are using as medicines. Some medicinal plants recorded in Wayu Tuka District were also used as remedies in other parts of Ethiopia. Accordingly, 51 medicinal plants were documented in [28]; 47 species in [30]; 41 species in [24]; 36 species in [25]; 33 species in [31]; 30 species in [26] and 15 species in [32,33]. The fact that some of the reported plants are having similar uses elsewhere can be considered as indication of their pharmacological effectiveness [31].

Among the families, Fabaceae was represented by 15 species (12%) followed by Solanaceae which had 8 species. The finding of the family Fabaceae as the contributor of higher number of plant species used for medicinal purposes than other families is in line with similar studies elsewhere in Ethiopia [30,31,34-36], whereas other researchers reported that Asteraceae is the leading family with highest number of medicinal plants [24,25,32]. Both findings are reasonable since the two families are both represented by higher number of species in the Ethiopian Flora.

The most widely used plant remedies by people of Wayu Tuka District were obtained from herbs which constituted the highest category of 55 species (43.6%). This finding is in line with other results [24,26,30,33,37]. Moreover, Giday *et al*. [33] reported that Zay people derive their medicine from herbs partly because of the fact that forests have been degraded and it takes much time and effort to harvest plant material from medicinal trees. It is true that herbs can grow everywhere (roadside, homegarden, farmland and in wild habitats) and common in the study area compared with other species such as trees, shrubs and climbers. However, other findings [25,27,34-36,38] indicated that shrubs were the most frequently used plant categories.

People of the study area, prepare remedies for human or livestock ailments, either from single plant or plant parts or by mixing them. Most of medicinal plants reported from the study area were claimed to be prepared from a single plant or plant part. Similar findings were also reported for use of multiple plants or plant parts for a single health problem [35,39,40] and use of single species was rare. This finding deviated from that reported by another researcher [30] who reported that 78% of the preparations of traditional medicine by people of Chelya Wereda were drawn from mixtures of different plants or plant parts and another work [36] also reported that local healers of Sokoru mostly used more than one plant species to prepare remedy for an ailment. In the present study, it was observed that healers mostly used multiple plants or plant parts in order to increase the strength and efficacy of the drug as they reported during the interview. For example, rabies was treated by mixing the bark of *Clausena anisata,* leaves of *Sida rhombifolia,* root of *Cucumis ficifolius,* and root bark of *Brucea antidysentrica*. They used different additives like soil, ash, honey, salt, sugar, local beer, milk and butter in order to increase the flavor, taste and general acceptability of certain orally administered remedies. This means that since traditional medicines could have sour or bitter tastes in most cases the additives reduce such tastes and may even improve the efficacy of the medicine.

The finding of leaves to be the most widely harvested plant parts is inline with other results [24,30-32]. However, other findings [25,26,35] indicated that roots were mostly utilized plant part. It was reported that collection of root, bark and whole plants might kill plants in harvest [41]. The same document also reported that root, which accounts for 58.3% is the most extensively used plant part in Ethiopia. Utilization of leaves may not cause detrimental effect on the plants compared with plant species in which root is utilized. However, this has to be seen on a case by case basis.

### Mode of preparation, condition and route of application

Crushing was the most widely used method of preparation of remedy in the area. This finding agrees with the findings of Getaneh [24], Yineger and Yehuwalaw [36] and Yirga [42]. However, the finding of Mesfin *et al*. [25] and Amenu [30] shows that powdering and pounding are the dominant method of preparation in Wonago and Chelya Woreda respectively.

The majority of the medicines (74%) were prepared from fresh plant materials in the study area. Different studies from other parts of Ethiopia also reported similar results [24-26,28,31,32,42,43]. Preference of application of fresh plant parts is related to the efficiency of the medicines in curing diseases compared with the dried parts. This is because of the fact that most important chemical may be changed upon drying [44]. On the other hand, utilization of fresh plant parts may threaten the plants through frequent collection including in dry seasons since local people made minimal efforts in storing dried plant material for later use.

About 64% of the medicines in the area were administered orally and 27.3% used as dermal applications. In similar studies, other researchers reported oral administration of medicine as the leading route of application, in particular the results from Chelya Wereda [30] and Fentalle area [35], which accounted for 60.3% and 54.7% respectively. Similarity among those results showed that internal diseases are more prevalent in Ethiopia.

### Effectiveness and dosage of medicines

Medicinal plants are reported to be effective in certain diseases. The results from Sokoru District [36] indicated that local people visit traditional healers even in preference to modern medications. In the present study, local people indicated their preferences for traditional medicines over modern drugs to get relief from certain diseases including rabies and health problems associated with the liver, spider poisoning and those caused by bat urine.

Lack of consistency regarding amount of medicines to be used was observed among informants during the interview. It was reported that lack of precise dosage is one drawback of traditional medicinal plants [39,45,46].

### Threats to medicinal plants and conservation practices in the study area

Medicinal plants are at increasing risk from destruction of their habitats (agricultural activities, fire wood collection, collecting plants for construction, overgrazing by domestic animals, urbanization) and over-harvesting of known medicinal species. As already indicated, most medicinal plants in the study area relied on collection of leaves and this practice helps to reduce the rate of threats on plant species compared with utilization of roots. However, there were medicinal plants in the study area in which roots were collected for treatment of ailments. As a result, over collection poses a threat to medicinal plants in the cases of harvesting the roots. This was observed in the cases of *Cucumis ficifolius*, *Rumex nepalensis* and *Securidaca longepedunculata.* These medicinal plant species were used to treat blackleg, evil eye and liver diseases and informants reported that it is difficult to collect them easily and they are getting lost due to over utilization for medicinal purposes.

Informants highly cited that deforestation became the most threatening factor on medicinal plants as reported by other researchers [25,33]. In this respect, plant species with multiple uses were said to be highly affected as also witnessed during the research. For instance, local people of the area preferred *Cordia africana* for construction; timber production, charcoal and medicine and it is on the verge of being eliminated from the area.

The conservation of medical plants in the study area was minimal rather beliefs have some contributions to conservation of medicinal plants as also reported by another study [27,30]. Keeping the knowledge on medicinal plants secretly can also have some contribution for their conservation. Thus, if medicinal plants are known by all people the impact could increase [25].

## Conclusion

The present study records 126 reported medicinal plants and their uses and majority of traditional medicinal plants were harvested mostly from wild. In the study area herbs constituted the highest proportion of medicinal plants to be utilized. Majority of medicinal plant species were harvested for their leaves and utilization of leaves may not cause detrimental effect on the plants compared with plant species in which root is utilized. Although high numbers of medicinal plants have been reported to be used for the treatment of human and livestock health problems, they are being threatened by different human activities while conservation efforts are minimal in the area. Deforestation for agricultural purpose was the major threat reported to medicinal plants of the study area. To save medicinal plants from further loss, the District Agricultural Office needs to team up with the local people, including by providing to the community planting materials of the most threatened and preferred medicinal and multipurpose species so that they can grow them in their homegardens. Moreover, the documented medicinal plants can serve as a basis for future investigation of modern drug.

## Competing interest

The authors declare that they have no competing interests.

## Authors’ contributions

We have made substantive intellectual contribution to this study in data collection, identification of plants, preparation and editing of the manuscript and proof reading. MM conducted the field work, identified the plants, analyzed the data and wrote the draft of manuscript. ZA identified the plants, edited the manuscript, provided comments and suggestions on the manuscript. EK identified the plants, provided comments and suggestions on the manuscript. AB and BW edited, provided comments and suggestions on the manuscript. All authors read and approved the final manuscript.
